# A Case of Syphilis Associated With Genital and Extragenital Hard Chancres, Inguinal Lymphadenitis, and Systemic Symptoms

**DOI:** 10.7759/cureus.72017

**Published:** 2024-10-21

**Authors:** Yoshihito Mima, Masako Yamamoto, Ken Iozumi

**Affiliations:** 1 Department of Dermatology, Tokyo Metropolitan Police Hospital, Tokyo, JPN

**Keywords:** erosion, extragenital, hard chancre, hematologic malignancy, lymphadenopathy, syphilis, ulcer

## Abstract

Syphilis is a sexually transmitted infection caused by the spirochete *Treponema pallidum* (TP) subspecies *pallidum*. Syphilis can be transmitted via contact with infected bodily fluids, such as blood or semen, congenital infection, blood transfusion, or organ transplantation. After a brief incubation period, the disease manifests with signs and symptoms such as genital ulcers, erythematous papules on the palms and soles, fever, and lymphadenopathy. The clinical presentation of syphilis is varied, making its diagnosis challenging. Syphilitic ulcers or erosions (hard chancres) may occur in extragenital regions, and the disease may present as isolated lymphadenopathy without skin lesions. In some cases, lymphadenopathy accompanied by elevated levels of inflammatory markers may mimic hematologic malignancies.

Herein, we report a rare case of syphilis associated with genital and extragenital chancres, as well as inguinal lymphadenopathy, in a 31-year-old man. The patient developed a finger ulcer and painless swelling of the left inguinal lymph node, initially suspected to be a skin infection and treated with topical and oral antibiotics, but with poor improvement. During treatment, systemic symptoms such as fever, malaise, joint pain, and loss of appetite emerged, and blood tests showed elevated C-reactive protein and soluble interleukin-2 receptor levels, suggesting suppurative lymphadenitis or hematologic malignancy. Although a lymph node biopsy was considered, a genital ulcer appeared later, raising suspicion of syphilis. TP antibodies were positive, and titers of quantitative rapid plasma reagin and TP antibodies were high, leading to a diagnosis of syphilitic chancres and lymphadenitis. After two months of amoxicillin treatment, the clinical symptoms and blood test results improved. This case highlights the potential time lag in the appearance of chancres in multiple regions and shows that syphilitic lymphadenopathy may mimic hematologic malignancies.

## Introduction

Syphilis is a sexually transmitted infection caused by the spirochete *Treponema pallidum* (TP) subspecies *pallidum*. The clinical manifestations arise from the local inflammatory response triggered by the spirochetes replicating within tissues [1]. TP, the bacterium responsible for syphilis, is transmitted through contact with infected bodily fluids, such as blood or semen, as well as via transplacental transmission, blood transfusion, or organ transplantation [2,3]. Coinfection with human immunodeficiency virus is also common, occurring in approximately 18% of cases [4]. Syphilis progresses with an incubation period ranging from 1 to 13 weeks. The disease course is divided into several stages: primary, secondary, and tertiary, each characterized by distinct features [2].

Patients with primary syphilis typically present around three weeks post-infection with a single ulcer or multiple lesions on the genitals or other areas of sexual contact, accompanied by regional lymphadenopathy [5]. Lymphadenopathy in syphilis most commonly affects the inguinal lymph nodes, although it can sometimes extend to the cervical or axillary lymph nodes [6]. While syphilitic lymphadenitis is generally painless, it may occasionally result in lymph node swelling and tenderness, often accompanied by fever and malaise [5,6].

Following the resolution of the primary lesion, secondary symptoms may emerge six to eight weeks later. Secondary syphilis presents with various cutaneous manifestations, such as maculopapular rashes, palmar-plantar rashes, and condyloma, in addition to systemic symptoms like fever and headache [7,8]. The generalized rash at this stage is highly infectious. Once these symptoms subside, patients enter a latent phase, which may last for several years. During the first one to two years of latency, patients are still considered infectious due to a 25% risk of relapse with secondary syphilis-like symptoms [5]. Historical data indicate that 15-40% of untreated individuals will progress to tertiary syphilis, which can manifest as destructive cardiovascular or neurological diseases, severe skin or visceral lesions, or bone involvement. More recent data suggest that tertiary syphilis may be less common today, likely due to the widespread use of antibiotics [9].

Penicillin remains the optimal antibiotic for treating all stages of syphilis [9]. In cases where penicillin cannot be used due to allergic reactions, alternative drugs - including doxycycline, third-generation cephalosporins such as ceftriaxone, and probenecid - are used [10]. Although patients with human immunodeficiency virus (HIV) respond less effectively to antibiotic treatment than those without HIV, in most cases, penicillin is effective when HIV treatment is administered concurrently, along with long-term follow-up [11].

The introduction of penicillin in the 1940s significantly reduced the number of patients with syphilis [1]. However, recently, the number of patients with syphilis has increased, particularly among individuals with a high frequency of sexual activity, leading to an increase in horizontal transmission [12]. TP can also be transmitted vertically from an infected mother to the fetus during pregnancy or childbirth, increasing the risk and prevalence of congenital syphilis among the children of patients of childbearing age [1]. Congenital syphilis can lead to infant death or stillbirth in approximately half of the cases [1]. Therefore, early and accurate diagnosis of syphilis is crucial to prevent the progression of the infection. However, the diverse range of skin manifestations and clinical courses of syphilis often makes its diagnosis challenging in the early stages [4]. Herein, we report a rare case of syphilis associated with multiple chancres at both genital and extragenital sites, along with inguinal lymphadenitis, which posed a diagnostic challenge at the initial presentation.

## Case presentation

A 31-year-old man with no notable medical history or regular medication presented with a finger ulcer and painless swelling of the left inguinal lymph node soon after visiting a dermatology clinic. A skin infection was suspected, and gentamicin ointment was administered for the ulcer, but the treatment was not effective after three weeks. Thus, oral doxycycline (100 mg/day) was initiated. After a week of doxycycline treatment, the swelling of the left inguinal lymph node persisted, accompanied by tenderness and fever. Therefore, the patient was referred to our hospital because of the possibility of hematologic malignancies.

**Figure 1:**
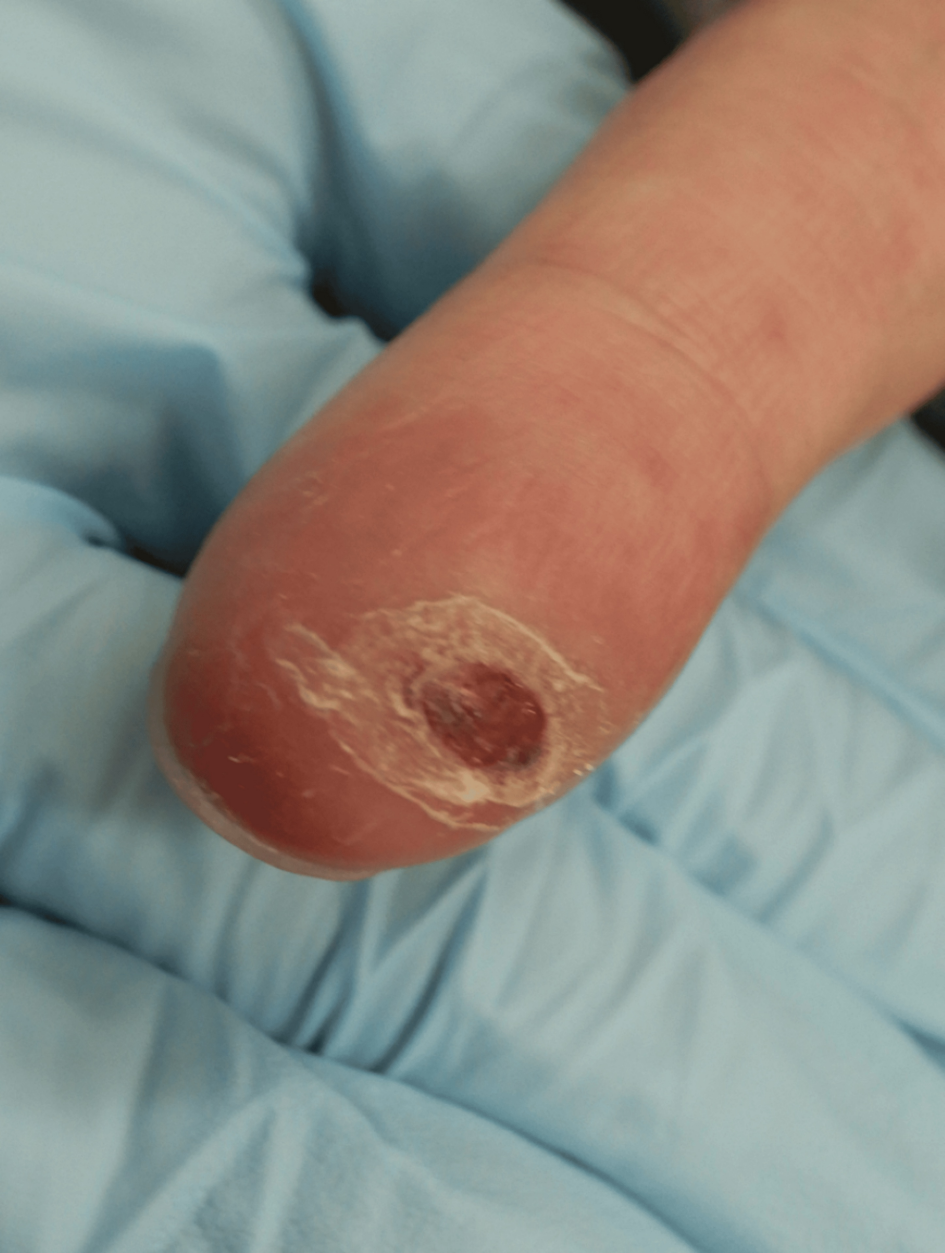
Skin ulcer on the right index finger

**Figure 2:**
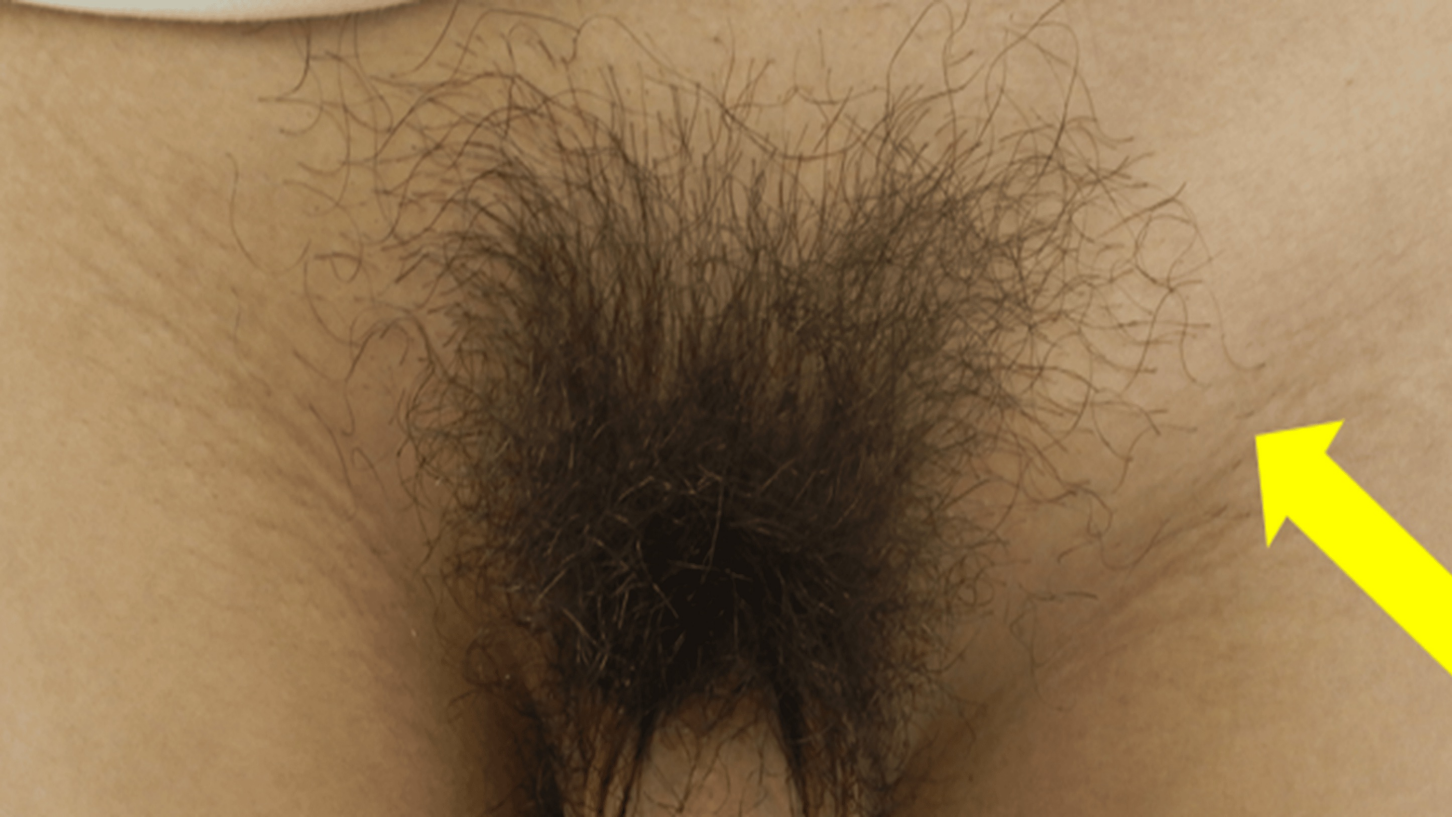
The left inguinal lymph node swelling (diameter, 4 cm; yellow arrow)

Physical examination revealed a finger ulcer (Figure 1) and painful swelling of the left inguinal lymph node (Figure 2). He had systemic symptoms, including fever, malaise, joint pain, and loss of appetite. No skin lesions other than the finger ulcer were detected. Moreover, no swollen lymph nodes other than the left inguinal lymph node were detected. The patient was married and had multiple sexual partners. He reported consistent condom use. Neither the patient nor his wife had a history of sexually transmitted infections. There was no history of intravenous drug use or blood transfusions.

Laboratory examinations at the beginning of systemic symptoms revealed elevated serum C-reactive protein (CRP) (4.0 mg/dL), immunoglobulin (Ig) G (2,424 mg/dL), IgA (611 mg/dL), IgM (233 mg/dL), and soluble interleukin-2 receptor (sIL-2R) (1,638 U/mL) levels. The T-cell Spot (T-SPOT) test was negative, and no abnormal blood cells, such as blasts or atypical lymphocytes, were detected in the peripheral blood.

**Figure 3:**
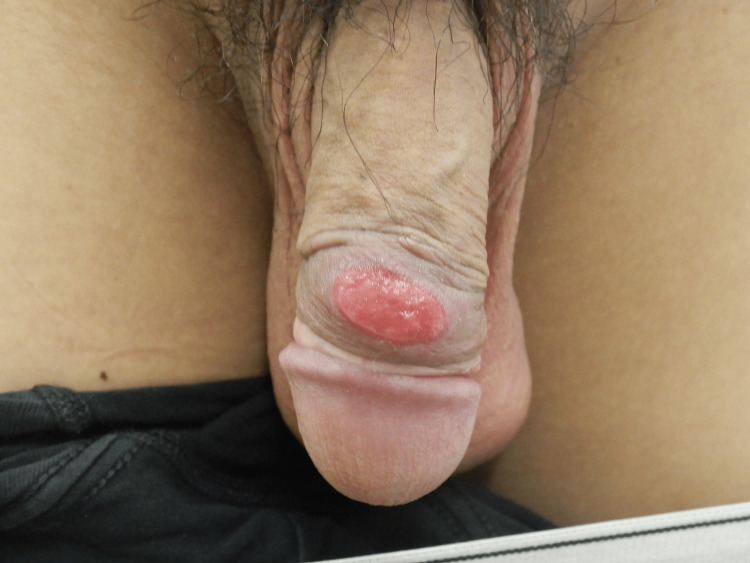
Skin erosion (diameter, 2 cm) on the penis

Given the systemic symptoms and laboratory findings, various diseases, such as suppurative lymphadenitis and hematologic malignancy, were considered differential diagnoses. At the follow-up visit, two weeks later, a skin erosion (diameter: 2 cm) was observed on the penis (Figure 3), raising the suspicion of syphilis.

Serum anti-TP antibodies were positive, with notably high titers of quantitative rapid plasma reagin (RPR) (292.6 R.U.) and TP antibodies (4,696 U/mL). Tests for other infections, including HIV, hepatitis B, and hepatitis C, were negative. All laboratory data from the first and second visits are shown in Table 1.

**Table 1:** Results of laboratory examination at the emergent of systemic symptoms RR: Reference range; AST: Aspartate aminotransferase; ALT: Alanine aminotransferase; Alk Phos: Alkaline phosphatase; LDH: Lactate dehydrogenase; CRP: C-reactive protein; ESR: Sedimentation rate; sIL2-R: soluble interleukin-2 receptor; IgG: Immunoglobulin G; IgA: Immunoglobulin A; IgM: Immunoglobulin M; HIV: Human immunodeficiency virus; T-SPOT: T-cell Spot; Ab: Antibody; RPR: Rapid plasma reagin; TP: Treponema; Hep: Hepatitis

Variable	Patient value	RR (adults)
AST	30 U/L	11-33 U/L
ALT	42 U/L	6-37 U/L
Alk Phos	101 U/L	35-104 U/L
Albumin	3.8 g/dL	3.8-5.0 g/dL
Total protein	8.2 g/dL	6.1-8.2 g/dL
Total bilirubin	0.7 mg/dL	0.2-1.2 mg/dL
LDH	156 U/L	135-214 U/L
CRP	4.00 mg/dL	<0.50 mg/dL
ESR	51 mm/hr	0-28 mm/hr
sIL2-R	1638 U/mL	200-580 U/mL
IgG	2424 mg/dL	870-1700 mg/dL
IgA	611 mg/dL	110-410 mg/dL
IgM	233 mg/dL	35-220 mg/dL
HIV 1/2 Ab, antigen	Nonreactive	Nonreactive
T-SPOT	Nonreactive	Nonreactive
RPR screen	Reactive	Nonreactive
TP antibody screen	Reactive	Nonreactive
Treponemal IgG+IgM Ab	Reactive	Nonreactive
Quantitative RPR level	292.6 R.U.	<1.0 R.U.
Quantitative TP antibody level	4696 U/mL	< 80 U/mL
Hep B Ab	Nonreactive	Nonreactive
Hep C Ab	Nonreactive	Nonreactive

Based on these findings, the patient was diagnosed with syphilitic left inguinal lymphadenitis and syphilitic chancres on the penis and right index finger. A lymph node biopsy was considered to confirm whether the lymphadenopathy was induced by syphilis; however, it was not performed due to its invasiveness and the patient’s preference for oral treatment. Consequently, oral amoxicillin (1,500 mg/day) was initiated.

**Figure 4:**
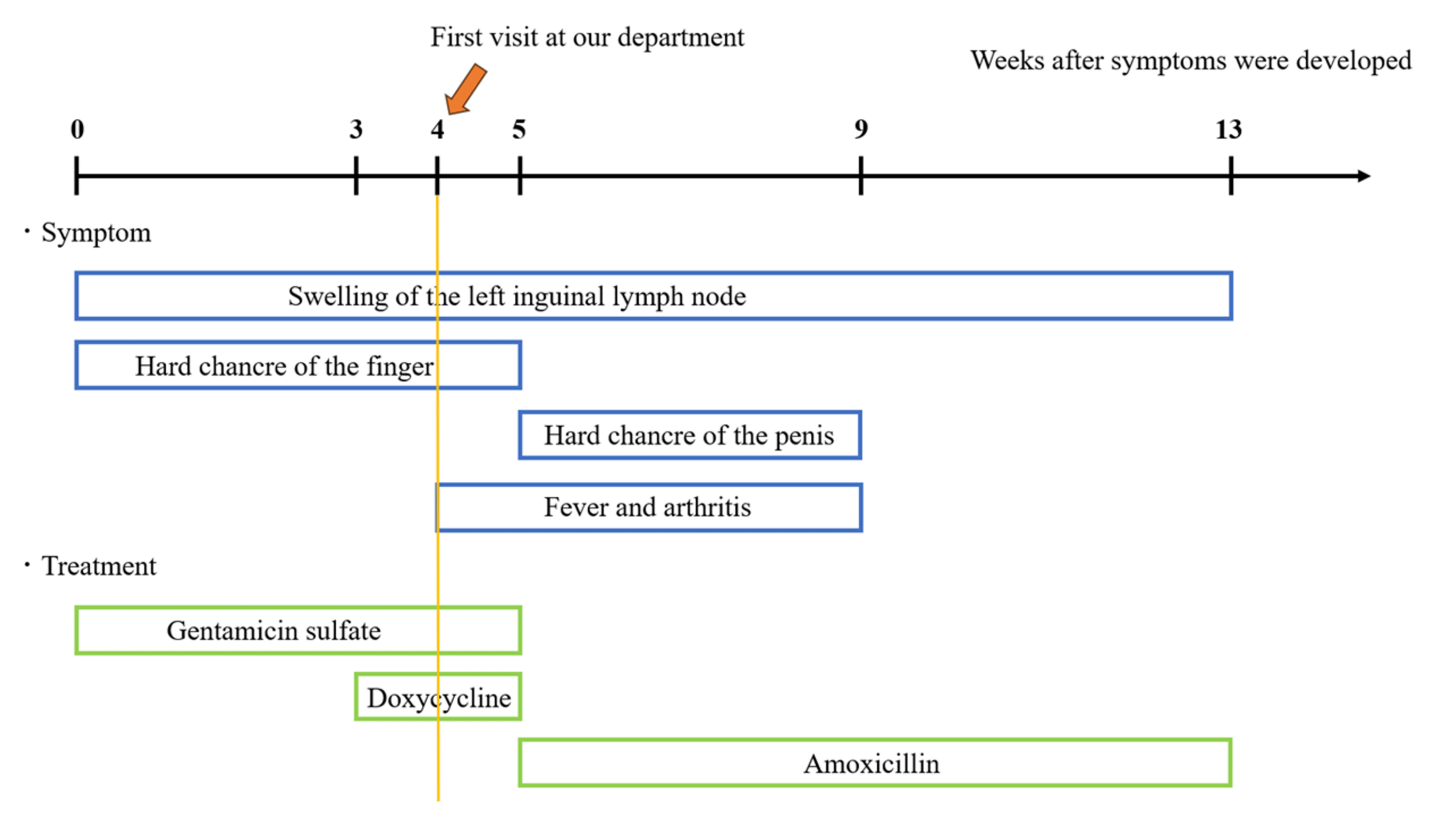
Course of the patient’s symptoms and treatments

After one month of treatment, the serum CRP level became normal, serum Ig levels improved, and the serum RPR level decreased by more than half of the original value (143.0 R.U.). The chancres on the finger and penis almost flattened, leaving pigmentation behind. Tenderness in the inguinal lymph nodes subsided, and palpation revealed a decreased size of the nodes (diameter: <1 cm). Although the serum CRP level had normalized and the serum RPR/LA level had decreased to half of the original value, the serum sIL-2R level remained elevated at 1,000 U/mL, and minimal swelling of the left inguinal node persisted. Therefore, oral amoxicillin treatment was extended. Two months after starting amoxicillin, all skin lesions and lymph node swelling and tenderness had completely resolved, and serum levels of all markers, including that of sIL-2R, normalized. Consequently, all treatments were discontinued. Based on the clinical findings and treatment course, the final diagnosis was syphilitic chancres on the penis and right index finger associated with syphilitic left inguinal lymphadenitis. The course of the patient’s symptoms and treatments is shown in Figure 4.

## Discussion

Herein, we report a rare case of syphilis manifesting as genital and extragenital chancres and inguinal lymphadenopathy. Initially, the patient presented with inguinal lymphadenopathy without accompanying inguinal ulcers or erosions, which made it difficult to consider syphilis as a differential diagnosis. The patient developed fever, malaise, and elevated serum CRP and sIL-2R levels, leading us to primarily consider suppurative lymphadenitis or hematologic malignancy. We contemplated whole-body computed tomography (CT) and lymph node biopsy; however, we ultimately decided against performing these investigations, and syphilis was diagnosed following the emergence of inguinal erosion.

Rapid lymph node enlargement typically raises concerns regarding the progression of hematologic lymphoma [13]. Other differential diagnoses include suppurative lymphadenitis, cat-scratch disease, tuberculous lymphadenitis, sarcoidosis, and metastatic malignancies [14]. When no skin manifestations are present, a lymph node biopsy, despite its invasiveness, may be necessary to diagnose these conditions [14].

A definitive pathological diagnosis of syphilis requires the detection of spiral microorganisms via Warthin-Starry staining or confirmation of TP antibody positivity via immunostaining [15]. Hematoxylin and eosin staining reveals typical histopathological findings of syphilitic lymphadenitis, including chronic inflammation and extensive fibrosis of the lymph node capsule and pericapsular tissues, sheets of plasma cells in the interfollicular areas, vascular changes such as phlebitis and endarteritis, and follicular hyperplasia [4]. However, syphilitic lymphadenitis can exhibit various pathological patterns, making it difficult to establish specific histopathological criteria [15]. In some cases, differentiating syphilitic lymphadenitis from suppurative lymphadenitis or sarcoidosis, based on pathological results, may be challenging [15]. Given the invasiveness of lymph node biopsy, reserving it for cases where syphilitic lymphadenitis is clinically difficult to diagnose may be prudent.

In some cases, such as the present case, inguinal syphilitic lymphadenitis may precede skin manifestations in the same region, or the disease may present with lymphadenitis alone, without any skin lesions [16]. When isolated inguinal lymphadenopathy is encountered, considering syphilis as a differential diagnosis may help avoid unnecessary invasive investigations.

Primary syphilis typically manifests as a single chancre in the genital region [17]. However, primary syphilitic lesions can occasionally occur in extragenital areas, such as the lips, tongue, conjunctiva, neck, abdomen, interscapular region, arms, palms, fingers, and thighs [17]. Because TP cannot penetrate intact skin or mucous membranes, it invades through small abrasions on the epidermis or mucosal epithelium [18]. Thus, syphilis can present with multiple chancres, including those in extragenital locations, if the skin or mucosa in these areas is compromised. Although the present case had no history of finger injury, syphilis might have been transmitted through an unnoticed lesion on the finger caused by injury, leading to the development of the chancre. When chancres appear on extragenital regions, as in the present case, early diagnosis is challenging. Although chancres can spontaneously resolve within weeks to months without treatment, early diagnosis is crucial to prevent progression to secondary syphilis during the latent phase [1]. In the present case, the chancre on the finger regressed after treatment with topical gentamicin and oral doxycycline. Doxycycline is a second-line treatment for syphilis and might have contributed to the regression [10]. However, even though the chancre on the finger resolved, the inguinal lymph nodes remained swollen, and penile erosions appeared, indicating the persistence of systemic infection and inflammation. Thus, the finger chancre might have spontaneously regressed independently of the oral doxycycline treatment [1]. In syphilis, short-term antibiotic use can potentially mask skin manifestations such as erosions or ulcers. Therefore, caution should be exercised when administering antibiotics for skin lesions or ulcers to avoid obscuring the clinical presentation of the disease.

We encountered a rare case of extragenital chancre and inguinal lymphadenitis preceding the appearance of genital erosion. Chancres can also appear in extragenital areas, sometimes with a time lag. Syphilitic lymphadenitis may occur on its own, without skin manifestations, or precede the appearance of skin lesions. The mechanisms behind these phenomena are not well understood and should be the subject of future investigation. Given that syphilis is often referred to as the “great imitator” owing to its diverse clinical presentations [1], considering syphilis as a differential diagnosis when encountering unexplained lymphadenitis or skin lesions such as ulcers or erosions in young patients is essential.

## Conclusions

The present case highlights that lymphadenitis can precede the emergence of skin lesions and that chancres can appear in multiple locations, including extragenital areas, with a time lag. Additionally, syphilitic lymphadenitis may present with elevated serum levels of inflammatory markers, mimicking hematologic lymphomas. Since syphilis has a wide range of clinical manifestations, considering it as a differential diagnosis when encountering unexplained lymphadenitis, ulcers, or erosions is essential, thus avoiding invasive tests such as CT scans or lymph node biopsies.

## References

[1] Giacani L, Lukehart SA (2014). The endemic treponematoses. Clin Microbiol Rev.

[2] Peeling RW, Mabey D, Kamb ML, Chen XS, Radolf JD, Benzaken AS (2017). Syphilis. Nat Rev Dis Primers.

[3] Bowen VB, Braxton J, Davis DW (2019). Sexually transmitted disease surveillance 2018. https://stacks.cdc.gov/view/cdc/79370.

[4] Kassem Youssef H, Blind A, Chouta Ngaha F, Drenou B, Nojavan H, Michel C (2018). Secondary pulmonary syphilis: case report and review of literature. Ann Dermatol Venereol.

[5] Golden MR, Marra CM, Holmes KK (2003). Update on syphilis: resurgence of an old problem. JAMA.

[6] Hartsock RJ, Halling LW, King FM (1970). Luetic lymphadenitis: a clinical and histologic study of 20 cases. Am J Clin Pathol.

[7] Barei F, Murgia G, Ramoni S, Cusini M, Marzano AV (2022). Secondary syphilis with extra-genital condyloma lata: a case report and review of the literature. Int J STD AIDS.

[8] Peeling RW, Mabey D, Chen XS, Garcia PJ (2023). Syphilis. Lancet.

[9] Wöhrl S, Geusau A (2007). Clinical update: syphilis in adults. Lancet.

[10] (2024). WHO guidelines for the treatment of Treponema pallidum (syphilis). https://www.who.int/publications/i/item/9789241549714.

[11] Rolfs RT, Joesoef MR, Hendershot EF (1997). A randomized trial of enhanced therapy for early syphilis in patients with and without human immunodeficiency virus infection. The syphilis and HIV study group. N Engl J Med.

[12] Ramchandani MS, Cannon CA, Marra CM (2023). Syphilis: a modern resurgence. Infect Dis Clin North Am.

[13] Jin A, Feng J, Wang Z, Jiang L, Hu Y, Zhao K, Huang H (2019). Severe dyspnea caused by rapid enlargement of cervical lymph node in a relapsed/refractory B-cell lymphoma patient following chimeric antigen receptor T-cell therapy. Bone Marrow Transplant.

[14] Charles RC, Sertic M, Neilan AM, Sohani AR (2021). Case 11-2021: a 39-year-old woman with fever, flank pain, and inguinal lymphadenopathy. N Engl J Med.

[15] Yorita K, Ito C, Fujioka A, Kashiwagi K, Yamai H, Nakatani K, Kumon T (2023). Primary syphilis presenting as a painful unilateral inguinal lymphadenopathy, without cutaneous manifestations, in a 71-year-old Japanese man: a case report. Radiol Case Rep.

[16] Bickford DD, Johnson P, Brahmbhatt N, Kroft S (2023). Cervical syphilitic lymphadenitis in a 29-year-old female: a case report. Cureus.

[17] Zheng S, Liu J, Xu XG, Gao XH, Chen HD (2014). Primary syphilis presenting as bilateral nipple-areola eczematoid lesions. Acta Derm Venereol.

[18] Fukuda H, Takahashi M, Kato K, Oharaseki T, Mukai H (2015). Multiple primary syphilis on the lip, nipple-areola and penis: an immunohistochemical examination of Treponema pallidum localization using an anti-T. pallidum antibody. J Dermatol.

